# Impact of COVID-19 Pandemic on Intervals and Outcomes of Repeated Transarterial Chemoembolization in Patients With Hepatocellular Carcinoma

**DOI:** 10.3389/fonc.2021.602700

**Published:** 2021-05-06

**Authors:** Zhi-Cheng Jin, Li Chen, Bin-Yan Zhong, Hai-Dong Zhu, Chu-Hui Zeng, Rui Li, Jin-He Guo, Shi-Cheng He, Gang Deng, Xiao-Li Zhu, Cai-Fang Ni, Gao-Jun Teng

**Affiliations:** ^1^ Center of Interventional Radiology and Vascular Surgery, Department of Radiology, Zhongda Hospital, Medical School, Southeast University, Nanjing, China; ^2^ Department of Interventional Radiology, The First Affiliated Hospital of Soochow University, Suzhou, China

**Keywords:** COVID-19, hepatocellular carcinoma, transarterial chemoembolization, follow-up interval, overall response rate

## Abstract

**Purpose:**

Given that the novel coronavirus disease (COVID-19) pandemic has disrupted operations globally, an institution’s ability to repeat transarterial chemoembolization (TACE) for patients with hepatocellular carcinoma (HCC) has also been affected. The aim of this study was to evaluate the impact of the COVID-19 on the intervals and outcomes of TACE in HCC patients.

**Materials and Methods:**

This retrospective study included 154 HCC patients who underwent follow-up after TACE treatment from January 2020 to March 2020 (n = 71, study group) and January 2019 to March 2019 (n = 83, control group) at two institutions in China. The endpoints included the follow-up interval and overall response rate (ORR). Multivariate logistic regression analyses were performed to identify independent risk factors for a worse ORR. The cut-off point was determined to divide follow-up durations into long- and short-intervals.

**Results:**

The median follow-up interval was 82.0 days (IQR, 61–109) in the study group, which was significantly longer than 66.0 days (IQR, 51–94) in the control group (*P* = 0.004). The ORR was 23.9 and 39.8% in the study and control group, respectively (*P* = 0.037). The cut-off value was 95 days. The grouping (OR, 2.402; 95% CI, 1.040–5.546; *P* = 0.040), long interval (OR, 2.573; 95% CI, 1.022–6.478; *P* = 0.045), and China liver cancer staging system (OR, 2.500; 95% CI, 1.797–3.480; *P <*0.001) were independent predictors for the efficacy of TACE treatment.

**Conclusions:**

The COVID-19 pandemic causes a longer follow-up interval in general, which may further lead to a lower ORR in HCC patients. Those with a follow-up interval of >95 days tend to have a worse prognosis.

## Introduction

The novel coronavirus disease (COVID-19) pandemic, caused by the severe acute respiratory syndrome coronavirus 2 (SARS-CoV-2), has affected more than 119,960,700 people from 223 countries as of March 16, 2021 (1). Medical centers are overwhelmed due to infection control and suffered a severe shortage of testing and personal protective equipment, making normal practice very challenging over the rapid spread of COVID-19 (2, 3). In such circumstances, cancer patients had to face their treatment either delayed or interrupted, and irreversible progression becomes a potential risk to their health (4–6). A nationwide analysis from China showed that cancer patients may be subjected to a higher risk of COVID-19 infection and worse prognosis compared with healthy individuals (7). With the necessity of balancing limited resources to protect public health, many healthcare centers have prioritized COVID-19 infection prevention and management protocols to a degree that has sacrificed routine operating procedures, and the management of HCC patients was expectedly affected (8–11).

Transarterial chemoembolization (TACE) is currently recommended as the standard therapy for intermediate stage HCC by the Barcelona Clinic Liver Cancer (BCLC) therapeutic strategy and has been the most widely used treatment modality for patients with unresectable HCC (12–14). The global HCC BRIDGE study showed that TACE was also widely utilized in patients with advanced HCC and patients with early-stage HCC who refuse or not amenable to resection (13, 15, 16). According to the China liver cancer staging (CNLC) system proposed in the Guidelines for Diagnosis and Treatment of Primary Liver Cancer in China (2019 Edition), TACE is indicated for patients at Ib- to IIIb-stages (equivalent to part of BCLC A and C stages and the entire B stage) with Child–Pugh class A or B liver function and an Eastern Cooperative Oncology Group (ECOG) score of 0–2 (17, 18). As a locoregional and palliative therapy, repeated TACE on-demand with varying intervals—ranging from one to over four months—may result in different outcomes and prognosis (19–22). Nevertheless, the influence of the COVID-19 pandemic on TACE follow-up intervals and outcomes remain unknown. The purpose of this study was to evaluate the impact of the COVID-19 on the intervals and outcomes of TACE in HCC patients.

## Materials and Methods

### Patients

Between January 1, 2020 and March 31, 2020, 71 consecutive HCC patients with follow-up data after TACE treatment were retrospectively screened for the study group at two institutions. Between January 1, 2019 and March 31, 2019, 83 consecutive patients from these two institutions constituted the control group. The study was approved by the institutional ethics review boards at the participating institutions and the requirement for written informed consent was waived due to its retrospective nature.

The inclusion criteria were: 1) 18 years or older; 2) diagnosed as Ib- to IIIb-stage HCC under the CNLC system of the 2019 Chinese guidelines (17, 18); 3) the ECOG performance scores ≤1; 4) Child–Pugh A or B liver function; and 5) received at least one TACE session before the study periods. The exclusion criteria were: 1) Child–Pugh C liver function or evidence of decompensated liver cirrhosis including refractory ascites, esophageal or gastric variceal bleeding, or hepatic encephalopathy; 2) enrollment in other clinical trials; 3) surgery or ablation performed after the latest TACE; 4) history of other concurrent malignancies; and 5) incomplete follow-up data.

### TACE Procedures

All patients received a standardized conventional TACE treatment performed by two experienced interventional radiologists (with >20 years of experience) following the Chinese clinical practice guidelines for TACE (23). The feeding arteries of the tumor was confirmed by digital subtraction angiography before superselective catheterization using a 2.6F microcatheter (Asahi Intecc CO., LTD, Akatsuki-Cho Seto, Aichi, Japan, and Boston Scientific, Marlborough, Massachusetts). An emulsion of mixtures of lipiodol (Lipiodol Ultra-Fluide; Guerbet, Roissy, France) and pirarubicin (Shenzhen Main Luck Pharmaceuticals Inc, Shenzhen, China) was injected, followed by the infusion of gelatin sponge particles (Hangzhou Alicon Pharm Sci&Tech CO., LTD). Under normal circumstances, TACE procedures were repeated “on-demand”: If residual viable tumors or intrahepatic recurrences were detected on the contrast-enhanced computed tomography (CT) or magnetic resonance imaging (MRI) taken every 4–8 weeks, the patient was evaluated for the feasibility of additional TACE treatment. Subsequent TACE procedures were to be discontinued if two or more consecutive disease progressions were observed on response evaluation CT/MRI.

### Assessments

The endpoint of this study included the follow-up interval and overall response rate (ORR). The follow-up interval was defined as the duration between the date of the latest TACE session before the two study periods and the date of follow-up in the two study periods. ORR was defined as the percentage of patients achieving either complete response (CR) or partial response (PR) according to the modified Response Evaluation Criteria in Solid Tumors (mRECIST) guideline (24). The baseline mRECIST status before the two study periods was evaluated, and the independent risk factors for the ORR were analyzed. Tumor size was defined as the diameter of the largest viable tumor.

### Statistical Analysis

Continuous variables were described as median with standard deviation and compared by Student’s *t*-test or non-parametric Mann–Whitney U test. Categorical variables expressed as frequencies (%) were compared by the Pearson χ^2^ test or Fisher exact test. Univariate and multivariate logistic regression analyses were performed to determine the predictors of ORR in all patients. Cut-off values of continuous data were determined using receiver operating characteristic (ROC) curve analysis by maximizing the Youden index for sensitivity and specificity optimization (25). A *P*-value of less than 0.05 was considered statistically significant. All statistical tests were carried out using SPSS (version 21.0; IBM, Somers, NY) and R software (version 3.63; R Project for Statistical Computing, http://www.r-project.org).

## Results

A total of 154 HCC patients were included in this study, with 71 in the study group and 83 in the control group. The patients received similar number of previous TACE sessions before the two study periods (3.0 ± 2.6 vs. 3.1 ± 2.5, *P* = 0.934). The baseline characteristics of all included patients were shown in **Tables 1** and **2**. The baseline mRECIST status of patients in the study group was superior to those in the control group (*P* = 0.028): PR, 24 (54.5%) *vs.* 14 (28%) patients; stable disease (SD), 12 (27.3%) *vs.* 18 (36%) patients; and progressive disease (PD), 8 (18.2%) *vs.* 18 (36%) patients. Twenty-seven patients of the study group and 33 of the control group received one session of previous TACE treatment with no baseline radiologic responses recorded.

**Table 1:** Patient characteristics of the two groups.

	Overall (n = 154)	Study group (n = 71)	Control group (n = 83)	*P* value
Sex				.010
Female	27 (17.5%)	6 (8.5%)	21 (25.3%)	
Male	127 (82.5%)	65 (91.5%)	62 (74.7%)	
Age (years)	62 (54–69)	60 (52.5–67)	62 (54–72)	.853
ECOG score				>.999
0	142 (92.2%)	65 (91.5%)	77 (92.8%)	
1	12 (7.8%)	6 (8.5%)	6 (7.2%)	
Hepatitis infection				>.999
absent	34 (22.1%)	16 (22.5%)	18 (21.7%)	
present (HBV/HCV)	120 (77.9%)	55 (77.5%)	65 (78.3%)	
Child–Pugh				.188
A	144 (93.5%)	64 (90.1%)	80 (96.4%)	
B	10 (6.5%)	7 (9.9%)	3 (3.6%)	
BCLC				.247
A	35 (22.7%)	16 (22.5%)	19 (22.9%)	
B	66 (42.9%)	35 (49.3%)	31 (37.3%)	
C	53 (34.4%)	20 (28.2%)	33 (39.8%)	
CNLC system^†^				.095
I b	35 (22.7%)	16 (22.5%)	19 (22.9%)	
II a	17 (11.0%)	6 (8.5%)	11 (13.3%)	
II b	50 (32.5%)	30 (42.3%)	20 (24.1%)	
III a	22 (14.3%)	6 (8.5%)	16 (19.3%)	
III b	30 (19.5%)	13 (18.3%)	17 (20.5%)	
ALB (g/L)	39.1 (35.8–43.2)	38.6 (34.6–41.6)	39.6 (36.2–43.9)	.191
TBIL (μmol/L)	15.3 (11.8–19.8)	16.4 (13.1–21.6)	13.8 (10.4–18.6)	.002
ALT (U/L)	30.7 (21.0–48.0)	29.2 (20.5–43.0)	33 (22.0–51.9)	.345
AST (U/L)	36 (27.0–49.8)	35 (27.0–45.5)	39.5 (27.2–56.3)	.292
AFP (ng/dl)				.081
<200	97 (63%)	51 (71.8%)	46 (55.4%)	
200–400	12 (7.8%)	3 (4.2%)	9 (10.9%)	
>400	45 (29.2%)	17 (24.0%)	28 (33.7%)	
Tumor number				.486
1	40 (26.0%)	17 (23.9%)	23 (27.7%)	
2	24 (15.6%)	9 (12.7%)	15 (18.1%)	
≥3	90 (58.4%)	45 (63.4%)	45 (54.2%)	
Tumor size (cm)	4.6 (2.7–7.4)	4.9 (2.5–7.0)	4.6 (2.9–8.1)	.534
Interval (days)	72.5 (55.8–102.0)	82 (61.0–109.0)	66 (51.0–94.0)	.004
Previous TACE sessions	3.0 (2.5)	3.0 (2.6)	3.1 (2.5)	.934
Vascular invasion				.176
absent	120 (77.9%)	59 (83.1%)	61 (73.5%)	
present	34 (22.1%)	12 (16.9%)	22 (26.5%)	
Extrahepatic metastases				>.999
absent	122 (79.2%)	56 (78.9%)	66 (79.5%)	
present	32 (20.8%)	15 (21.1%)	17 (20.5%)	

Data are median (IQR), n (%), or mean (SD). ECOG, Eastern Cooperative Oncology Group; BCLC, Barcelona Clinic Liver Cancer; AFP, alpha-fetoprotein; ALB, albumin; TBIL, total bilirubin; ALT, alanine transaminase; AST, aspartate transaminase. ^†^the China liver cancer staging (CNLC) system.

**Table 2:** Patient characteristics of the two institutions.

	Overall (n = 154)	Institution Ⅰ (n = 84)	Institution Ⅱ (n = 70)	*P* value
Sex				.742
Female	27 (17.5%)	16 (19.0%)	11 (15.7%)	
Male	127 (82.5%)	68 (81.0%)	59 (84.3%)	
Age (years)	62 (54–69)	61.5 (54–70)	62 (54–67)	.501
ECOG score				.217
0	142 (92.2%)	80 (95.2%)	62 (88.6%)	
1	12 (7.8%)	4 (4.8%)	8 (11.4%)	
Hepatitis infection				>.999
absent	34 (22.1%)	19 (22.6%)	15 (21.4%)	
present (HBV/HCV)	120 (77.9%)	65 (77.4%)	55 (78.6%)	
Child–Pugh				.976
A	144 (93.5%)	78 (92.9%)	66 (94.3%)	
B	10 (6.5%)	6 (7.1%)	4 (5.7%)	
BCLC				.079
A	35 (22.7%)	24 (28.6%)	11 (15.7%)	
B	66 (42.9%)	30 (35.7%)	36 (51.4%)	
C	53 (34.4%)	30 (35.7%)	23 (32.9%)	
CNLC system^†^				.050
Ib	35 (22.7%)	24 (28.6%)	11 (15.7%)	
IIa	17 (11.0%)	9 (10.7%)	8 (11.4%)	
IIb	50 (32.5%)	22 (26.2%)	28 (40.0%)	
IIIa	22 (14.3%)	16 (19.0%)	6 (8.6%)	
IIIb	30 (19.5%)	13 (15.5%)	17 (24.3%)	
ALB (g/L)	39.1 (35.8–43.2)	38.4 (35.5–40.9)	40.0 (36.1–45.1)	.017
TBIL (μmol/L)	15.3 (11.8–19.8)	14.5 (11.5–19.9)	15.4 (12.8–19.8)	.754
ALT (U/L)	30.7 (21.0–48.0)	32.0 (22.3–48.0)	29.6 (18.3–48.2)	.691
AST (U/L)	36 (27.0–49.8)	37.0 (28.0–50.8)	35.6 (26.0–50.4)	.392
AFP (ng/dl)				.953
<200	97 (63.0%)	53 (63.1%)	44 (62.9%)	
200–400	12 (7.8%)	7 (8.3%)	5 (7.1%)	
>400	45 (29.2%)	24 (28.6%)	21 (30%)	
Tumor number				.074
1	40 (26.0%)	28 (33.3%)	12 (17.1%)	
2	24 (15.6%)	12 (14.3%)	12 (17.1%)	
≥3	90 (58.4%)	44 (52.4%)	46 (65.8%)	
Tumor size (cm)	4.6 (2.7–7.4)	4.6 (2.4–8.0)	5.1 (3.0–7.0)	.624
Vascular invasion				.710
absent	120 (77.9%)	64 (76.2%)	56 (80%)	
present	34 (22.1%)	20 (23.8%)	14 (20%)	
Extrahepatic metastases				.115
absent	122 (79.2%)	71 (84.5%)	51 (72.9%)	
present	32 (20.8%)	13 (15.5%)	19 (27.1%)	
Grouping				>.999
study group	83 (53.9%)	45 (53.6%)	38 (54.3%)	
control group	71 (46.1%)	39 (46.4%)	32 (45.7%)	

Data are median (IQR), n (%), or mean (SD). ECOG, Eastern Cooperative Oncology Group; BCLC, Barcelona Clinic Liver Cancer; AFP, alpha-fetoprotein; ALB, albumin; TBIL, total bilirubin; ALT, alanine transaminase; AST, aspartate transaminase, ^†^the China liver cancer staging (CNLC) system.

**Figure 1:**
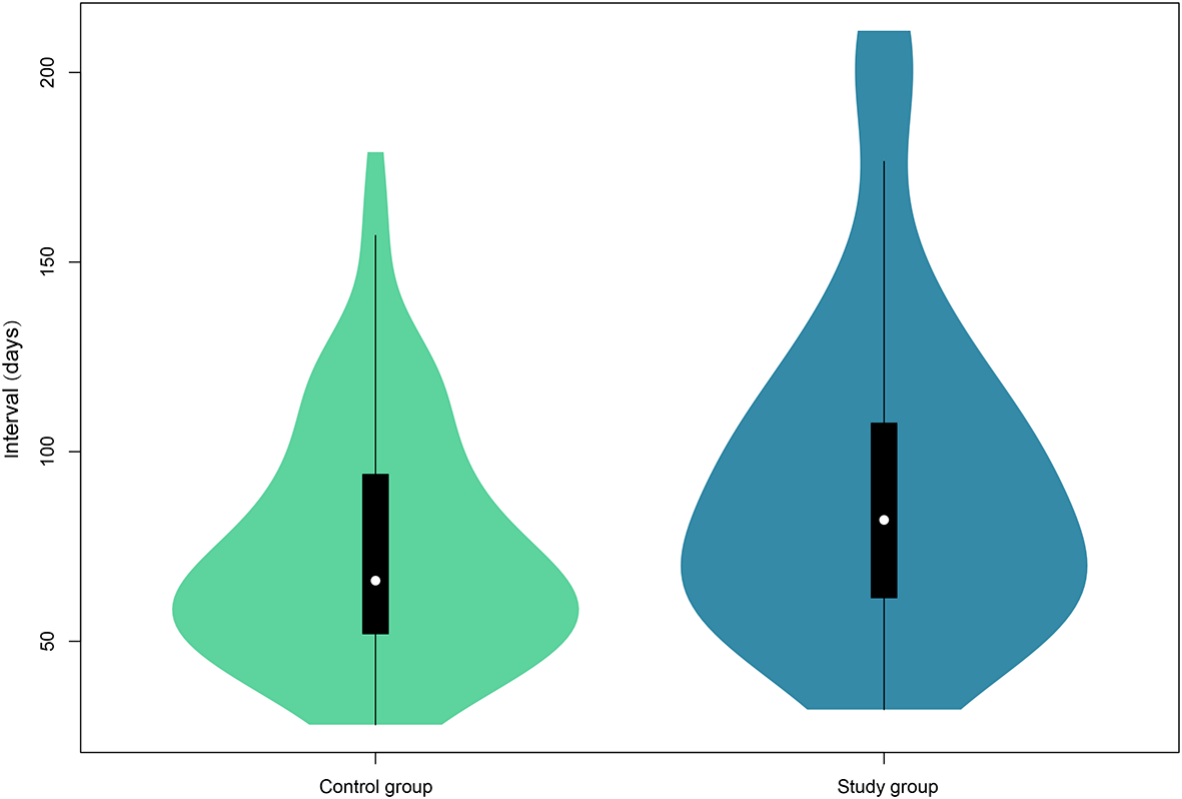
Violin plots of the follow-up interval of the two groups. The white dot indicates the median of the interval, whereas the black box presents the interquartile range. The thin black line shows 95% confidence interval. The width of the violin represents frequencies of interval values.

As shown in **Figure 1**, the median follow-up interval of patients in the study group (82.0 days, IQR, 61–109) was significantly longer than that of the control group (66.0 days, IQR, 51–94) (*P* = 0.004).

The radiologic responses of the two groups are shown in **Table 3**. The ORR after the latest TACE treatment was 23.9% (17/71) in the study group while being 39.8% (33/83) in the control group. The ORR of the patients in the pandemic era was significantly lower than that of the patients in the control group (*P* = 0.037).

**Table 3:** Radiologic response and ORR of the two groups.

Radiologic response	Study group (n = 71)	Control group (n = 83)	*P* value
CR	2 (2.8%)	7 (8.4%)	.111^*^
PR	15 (21.1%)	26 (31.3%)	
SD	25 (35.2%)	18 (21.7%)	
PD	29 (40.8%)	32 (38.6%)	
ORR	23.9%	39.8%	.037^#^

CR, complete response; PR, partial response; SD, stable disease; PD, progressive disease; ORR, overall response rate; ^*^Fisher exact test was used. ^#^Pearson χ^2^ test was used.

**Figure 2:**
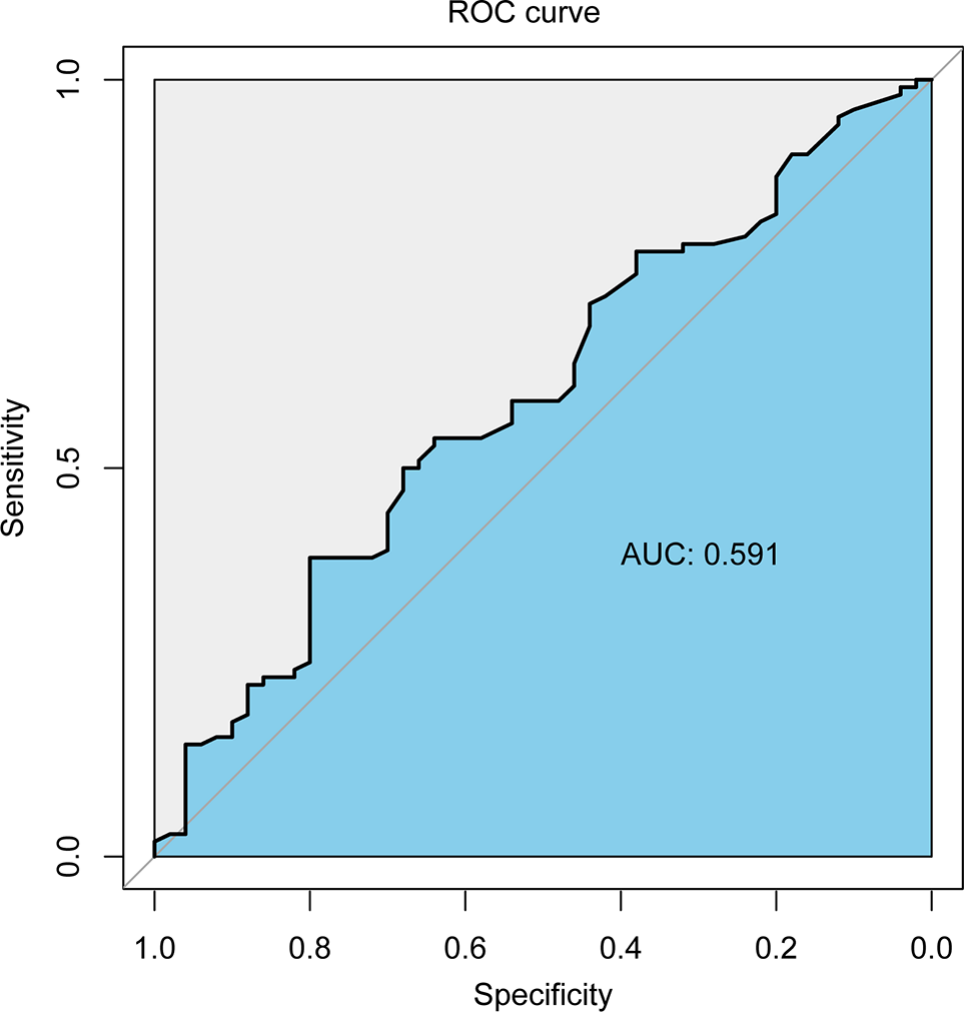
The receiver operating characteristic curve of the follow-up interval for predicting ORR of all patients.

**Figure 3:**
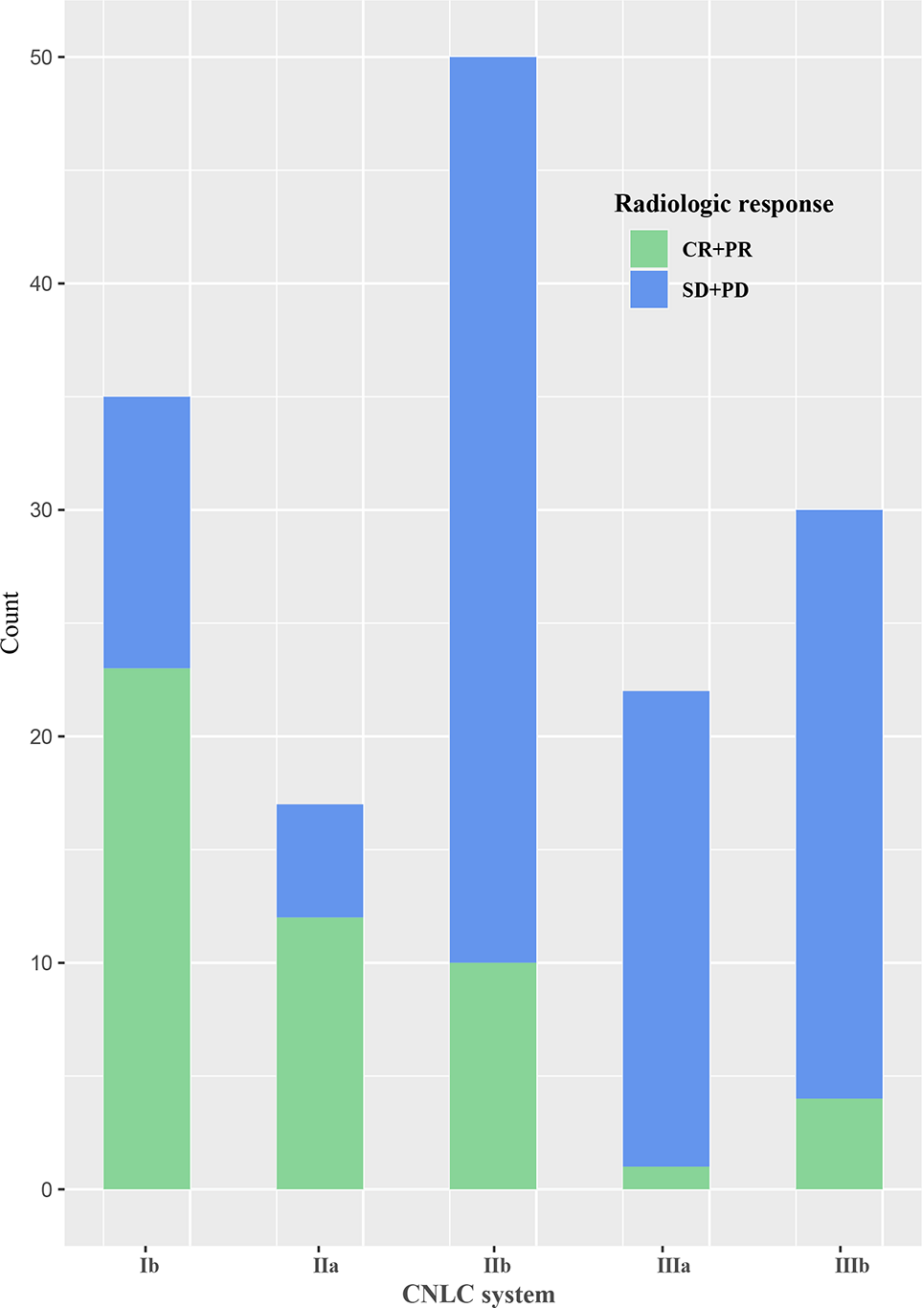
Bar graph of the distribution of radiologic response stratified by the China liver cancer staging (CNLC) system.

The area under the receiver operating characteristic curve of the follow-up interval for predicting ORR of all patients was 0.591 (**Figure 2**). Based on the ROC curve, the cut-off value to divide the follow-up interval into long- and short-intervals was determined to be 95 days. Univariate analyses showed that the tumor number (*P <*0.001), vascular invasion (*P* = 0.002), extrahepatic metastases (*P* = 0.028), BCLC stage (*P <*0.001), CNLC system (*P <*0.001), grouping (*P* = 0.038), and long interval (*P* = 0.024) were significantly associated with a poor ORR. After multivariate logistic regression analyses, the grouping, long interval, and CNLC system were selected as the independent predictors of the efficacy of TACE treatment (**Table 4**). The distribution of radiologic responses stratified by the CNLC system is presented in **Figure 3**.

**Table 4:** Uni- and multivariate analysis of prognostic factors for ORR.

	Univariate Analysis			Multivariate Analysis		
	OR	95% CI	*P* value	OR	95% CI	*P* value
AFP	1.424	0.954–2.124	.084			
Tumor number	1.548	1.262–1.898	<.001			
Vascular invasion	10.667	2.442–46.601	.002			
Extrahepatic metastases	3.156	1.135–8.775	.028			
BCLC	4.223	2.413–7.389	<.001			
CNLC system^†^	2.397	1.737–3.308	<.001	2.500	1.797–3.480	<.001
Grouping	2.096	1.041–4.223	.038	2.402	1.040–5.546	.040
Long interval	2.500	1.126–5.551	.024	2.573	1.022–6.478	.045

Multivariate logistic regression analysis was done with stepwise forward selection. ORR, overall response rate; OR, odd ratio; AFP, alpha-fetoprotein; BCLC, Barcelona Clinic Liver Cancer. ^†^The China liver cancer staging (CNLC) system.

## Discussion

In Jiangsu Province where the current study was conducted, the first-level public health reaction was initiated in late January following the first case of COVID-19 patient confirmed in mid-January, 2020. The routine follow-up of cancer patients may be delayed or interrupted on that occasion. With the spread halting of the virus, sporadic new domestic cases were reported at the end of March 2020. Both institutions involved in this study had restored operations to almost full capacity by April. However, the Lunar New Year holiday was an influencing factor that could prolong the follow-up interval for patients. To minimize the influence of the 7-day Lunar New Year holidays (February 5 to 12 in 2019 and January 25 to February 1 in 2020), the study periods covered the whole months of January to March. Thus, patients between January 2020 to March 2020 were included as the study group, while those treated one year earlier were included for comparison.

Our study demonstrated that the outbreak of COVID-19 indeed affected the follow-up intervals. The longer follow-up interval of the study group (82.0 days *vs.* 66.0 days; *P* = 0.004) brought a lower ORR (23.9% *vs.* 39.8%, *P* = 0.037), though these patients had a more favorable baseline mRECIST status compared to the control group (*P* = 0.028). Interestingly, there is no significant difference in the mRECIST status between the two groups within the study periods. Compared with the control group, the percentage of CR and PR in the study group was decreased while an increase was seen in the percentage of SD and PD, and this finding was contrary to the distribution of the baseline mRECIST status. This inverse relationship also implied that the longer follow-up interval caused by the COVID-19 further led to worse tumor responses in HCC patients.

By maximizing the Youden index of ROC analyses, the best cut-off point of the follow-up interval was determined as 95 days. Although the point was determined based on both groups, more patients of the study group were in fact followed up beyond 95 days. TACE repeated on-demand was proposed to offer more benefits than regular repetition (26, 27). Since then, this conception has been gradually accepted by several panels such as Expert Panel Opinion on Interventions in Hepatocellular Carcinoma (EPOIHCC) (19). However, the follow-up interval of repeated TACE “on-demand” are not yet clearly defined in most guidelines and can be multifarious in the practice. A cross-sectional survey on TACE techniques, with 1,160 respondents from 62 countries, showed that the typical clinical follow-up interval ranged from 2 weeks to 2 months (22). Among them, 46.1% (502/1,088) of respondents were likely to follow up with patients between 1 and 2 months, while 37.9% (412/1,088) were prone to follow up with patients between 2 weeks and 1 month. Very few of them followed up with patients beyond 2 months (8.3%, 90/1,088). Therefore, the present study might be the first to conclude 95 days as a cut-off point of follow-up intervals.

The appropriate length of interval for a repeated TACE is still controversial. In Yang and colleagues’ report, the long interval between the first two TACE sessions (>48 days) generated a better overall survival than a short interval (≤48 days) in HCC patients at BCLC stage-C, while no significant difference was found in patients at BCLC stage-A or B (21). Kim et al. demonstrated that patients undergoing repeated on-demand TACE with a shorter interval (<60 days) showed similar survival outcomes with those having conventional TACE intervals (>60 days) despite two different management policies were applied (physician A: median interval = 70 days, IQR = 61–82; physician B, median interval = 42 days, IQR = 36–55) (20). Of note, however, the definition of follow-up interval in our investigation was different from the one described in the studies above, which focused on the interval between the first two TACE sessions. Besides, sticking to the same clinical practice guidelines, TACE was performed by experienced operators with similar TACE formula and follow-up protocol in our study. Therefore, the results of our study could provide reference value for the operators on decision making, especially when the COVID-19 poses a risk to the public health (28).

The CNLC system largely differs from the BCLC staging and treatment strategy, which is more coherent with the actual practice in China (17, 18). In this guideline, TACE is indicated for stage IIb (more than three lesions and without vascular invasions/extrahepatic spread; equivalent to BCLC B), stage IIIa (with macrovascular invasion; equivalent to BCLC C), and stage IIIb (extrahepatic metastasis; equivalent to BCLC C) when the patients present with Child–Pugh class A or B liver functions and ECOG scores of 0–2. For patients at stage Ib (solitary lesion >5 cm and two to three tumors ≤3 cm; equivalent to BCLC-A) and IIa (two to three tumors ≥3 cm; equivalent to BCLC-B) who are unable or unwilling to undergo surgery, TACE becomes their option. In our study, as an independent predictor of ORR, the CNLC system gives better stratification of HCC patients than BCLC system does.

Our study has several limitations. First, it is a retrospective study with a relatively small sample size. Second, our study only included patients at two institutions in Jiangsu Province where is mildly affected by the COVID-19 pandemic, and therefore the results may not be applicable to patients in areas of higher disease prevalence in China or the world. Third, survival outcomes were not included in the study due to the limited study periods.

In conclusion, the COVID-19 pandemic results in a longer follow-up interval which may further lead to a lower ORR for HCC patients. Those with a follow-up interval of >95 days may experience a worse prognosis. However, additional studies with a larger population are required to validate our findings.

## Data Availability Statement

The original contributions presented in the study are included in the article/supplementary material. Further inquiries can be directed to the corresponding authors.

## Ethics Statement

The studies involving human participants were reviewed and approved by the Ethics Review Committee of the Zhongda Hospital and the Ethics Review Committee of the First Affiliated Hospital of Soochow University. The ethics committee waived the requirement of written informed consent for participation.

## Author Contributions

Z-CJ, LC, B-YZ, and H-DZ contributed to study design, data collection, data analysis, and drafting of the manuscript. C-HZ contributed to critical appraisal of the manuscript. RL contributed to data analysis and interpretation. J-HG, S-CH, GD, and X-LZ contributed to study design and critical appraisal of the manuscript. G-JT and C-FN finalized the manuscript and had final responsibility for the decision to submit for publication. All authors contributed to the article and approved the submitted version.

## Funding

This study was supported by the National Key Research and Development Project of China (2018YFA0704100), the National Natural Science Foundation of China (Major Scientific Research Instrument Development Program 81827805, 81441054, 81520108015, 81671796, 81901847), Jiangsu Provincial Medical Youth Talent Program (ZDRCA2016078), the Key Research and Development Project of Jiangsu Province (BE2019750), the Natural Science Foundation of Jiangsu Province (BK20190177), Innovation Platform of Jiangsu Provincial Medical Center (YXZXA2016005), and the Suzhou Science and Technology Youth Plan (KJXW2018003).

## Conflict of Interest

The authors declare that the research was conducted in the absence of any commercial or financial relationships that could be construed as a potential conflict of interest.

The reviewer YL declared a shared affiliation, with no collaboration, with several of the authors B-YZ, X-LZ, and C-FN to the handling editor at the time of the review.
